# A review of dietary patterns and the colorectal polyp-to-carcinoma sequence: polyp occurrence, polyp recurrence, and colorectal cancer

**DOI:** 10.3389/fnut.2026.1791951

**Published:** 2026-04-10

**Authors:** Changwei Huang, Beibei Sun, Yue Song, Lin Jiang, Ailing Gong, Tongxuan Zhu, Jintao Guo, Siyu Sun

**Affiliations:** 1 Department of Gastroenterology, Shengjing Hospital of China Medical University, Shenyang, Liaoning, China; 2 Engineering Research Center of Ministry of Education for Minimally Invasive Gastrointestinal Endoscopic Techniques, Shengjing Hospital of China Medical University, Shenyang, Liaoning, China

**Keywords:** colorectal cancer, dietary pattern, endoscopy, intestinal adenoma, precancerous lesion

## Abstract

Colorectal cancer (CRC) is the third most common cancer and the second leading cause of cancer-related deaths; it mostly arises from adenomatous and serrated polyps. The role of dietary patterns in the colorectal polyp-to-carcinoma sequence has attracted considerable attention. Diets high in vegetables, fruits, and fibres, as reflected in *a priori* healthy diet indices, such as the Mediterranean diet score or empirically derived prudent dietary patterns, are consistently associated with a reduced risk of polyp occurrence, and CRC. Conversely, unhealthy diets rich in red and processed meats, refined carbohydrates, and fats are associated with increased polyp occurrence and CRC risk. Epidemiological findings are consistent with mechanism-based indices, such as the Dietary Inflammatory Index. However, evidence linking dietary patterns to polyp recurrence remains comparatively limited. Taken together, the available literature suggests associations between dietary patterns and the polyp-to-carcinoma sequence and supports the rationale for the evaluation of dietary modification as a potentially preventive approach. Because most evidence is observational, well-designed prospective studies, preregistered long-term dietary intervention trials, and mechanistic investigations are needed to clarify causality and to quantify potential effects.

## 1 Introduction

**Figure 1:**
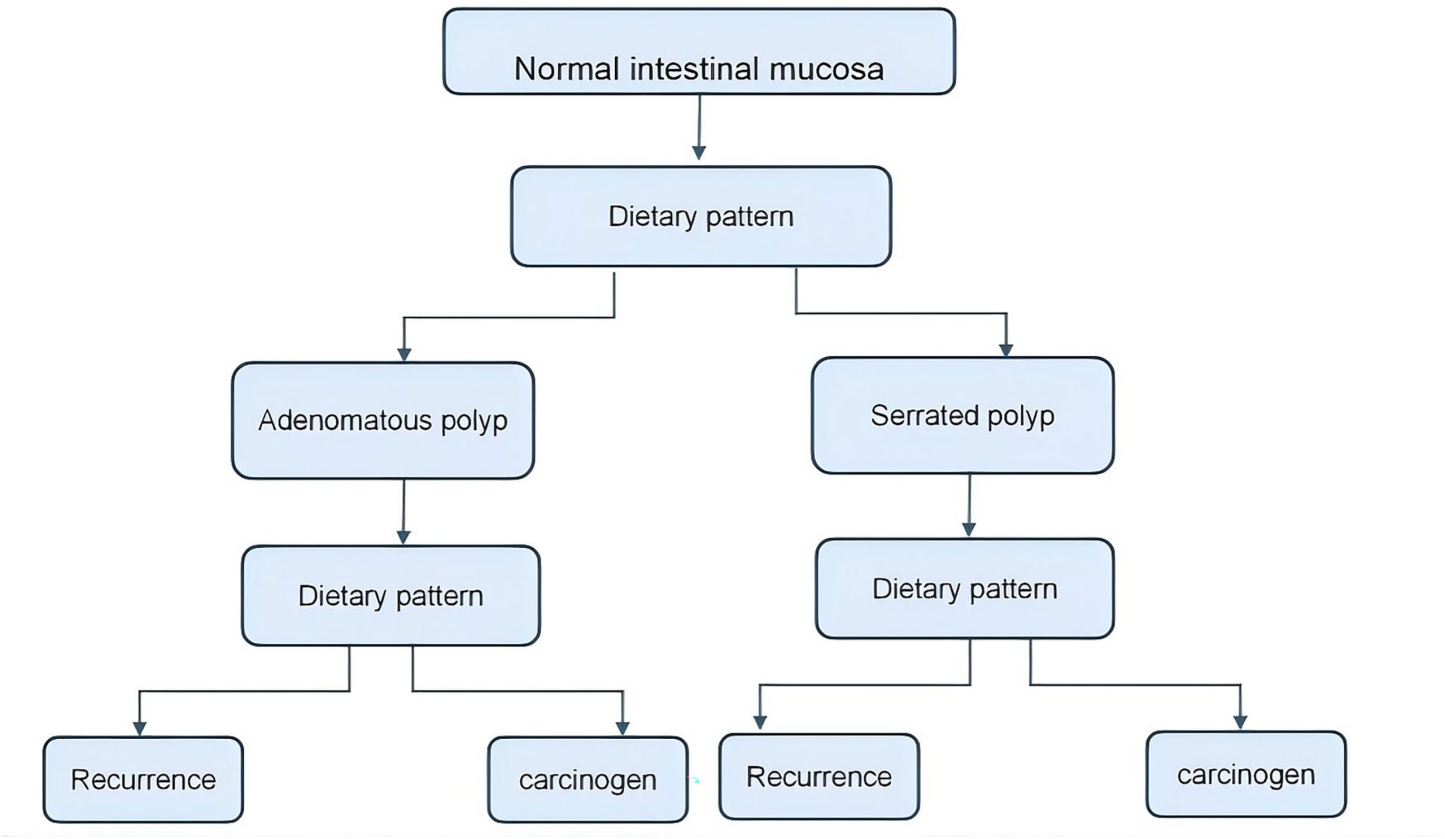
Schematic diagram illustrating dietary patterns mediating the occurrence of intestinal polyps, their recurrence, and malignant transformation.

Colorectal cancer (CRC) is highly prevalent malignancy worldwide, ranked as the third most commonly diagnosed cancer and the second leading cause of cancer-related mortality. Most CRCs originate from precancerous lesions, which are primarily adenomatous and serrated polyps. Adenomatous polyps represent the classic precursor lesions and are estimated to account for 85–90% of sporadic CRC (1). These polyps progress through the well-characterised adenoma–carcinoma sequence, whereby the normal colonic epithelium gradually transforms into carcinoma via intermediate adenomatous stages (referred to as adenomas). A significant subgroup of CRCs develops in parallel through the serrated pathway. Serrated polyps constitute a heterogeneous group that includes hyperplastic polyps, sessile serrated adenomas (SSA), and traditional serrated adenomas, which together form an alternative route to malignancy. In this pathway, progression is thought to be initiated by a benign hyperplastic polyp, which might evolve into an SSA and ultimately transform into carcinoma. Serrated lesions are believed to account for a minority of CRC cases (10–15%). Similar to adenomas, serrated precursors generally require several years to progress to cancer (referred to as serrated adenomas). Both adenomatous and serrated pathways involve identifiable benign lesions that precede malignancy, creating opportunities for therapeutic polyp removal. Even after the initial polyp is resected, patients retain the risk of developing new polyps over time. Clinical studies indicate that approximately 15–30% of patients develop one or more new polyps within a few years following polyp removal (2) (Figure 1).

With the early diagnosis of CRC possible, focus on lifestyle-related risk factors associated with the occurrence and recurrence of colorectal polyps has become important. Identifying and modifying lifestyle risk factors, such as smoking, alcohol consumption, and diet, are highly relevant for the prevention of CRC. Consequently, along with endoscopic surveillance, optimising modifiable risk factors, particularly diet, is a promising preventive strategy (3). Diet is a modifiable component in colorectal polyps. While traditional nutrition research focuses on individual nutrients or foods, it overlooks the complex effects of diet as a whole. Therefore, recent investigations have shifted focus toward examining dietary patterns to better capture the overall diet quality and its health impacts (4). Dietary pattern analyses can be *a priori*, based on predefined scores reflecting adherence to dietary guidelines or cultural diets, a posteriori, involving data-driven patterns identified through statistical techniques such as factor analysis, or biomarker-based, constructed around biological effects or markers such as inflammatory potential (5). Despite growing research on diet and colorectal tumours, a knowledge gap still persists regarding integrating the findings across the complete polyp-to-carcinoma sequence. Most existing reviews have addressed dietary patterns and CRC without systematically connecting them to the polyp stage. No comprehensive review has yet examined the correlation of dietary pattern with initial polyp formation through recurrence to the endpoint of CRC.

## 2 Methods

### 2.1 Literature search and study selection

The PubMed, Web of Science, and Embase databases were searched to identify all studies (published and unpublished) on dietary patterns with the colorectal polyp-to-carcinoma sequence. The search covered each database from inception to 31 December 2025. We searched databases using a combination of Medical Subject Heading terms and keywords related to dietary patterns (such as prior dietary patterns, posteriori dietary patterns, and mechanism-based dietary indices), colorectal adenomas, serrated colorectal adenomas, polyp occurrence, polyp recurrence, and colorectal cancer. Reference lists of eligible articles and relevant reviews were also screened to identify additional studies.

We included meta-analyses, randomised controlled trials, and observational studies (including prospective cohort, retrospective cohort, case–control, and cross-sectional studies) that examined *a priori* dietary indices, a posteriori dietary patterns, or mechanism-oriented dietary indices in relation to at least one outcome of interest: polyp occurrence, polyp recurrence, or CRC. Studies were classified as evaluating polyp occurrence if colorectal polyps were identified at an index colonoscopy in participants without documented prior polyps (including screening or diagnostic colonoscopy) or if participants had a history of endoscopic treatment for polyps (endoscopic mucosal resection [EMR] or endoscopic submucosal dissection [ESD]) and the outcome was metachronous polyps detected on surveillance colonoscopy after the baseline procedure. Exclusion criteria for this review were articles with incorrect exposure or design and studies published in non-English languages (6).

## 3 Results

### 3.1 Dietary patterns and the occurrence and recurrence of intestinal polyps

#### 3.1.1 A priori dietary patterns and the occurrence of intestinal polyps

Studies have indicated that adherence to predefined dietary patterns is associated with a decreased risk of colorectal polyps. For example, the Mediterranean diet, which is rich in fruits, vegetables, whole grains, legumes, olive oil, and fish, has been inversely linked to the occurrence of adenomas. An Israeli case–control study reported that individuals with a higher consumption of Mediterranean diet components had 8% lower odds of developing advanced adenomas than those with low adherence (7). Dose-responsive protection was observed in both advanced and serrated adenomas, whereas no significant association was observed in non-advanced adenomas. Interestingly, a large-scale study found that the Mediterranean diet was negatively associated with both adenoma and advanced adenoma formation (8). Similarly, this study found that participants who followed official dietary guidelines or the Dietary Approaches to Stop Hypertension (DASH) diet, which is rich in vegetables, fruits, whole grains, and low-fat dairy products, had a 20–30% lower incidence of colorectal adenomas (8) (Table 1). Although the protective effect was strongest in men, it was also evident among women who were non-obese or non-smokers. Further, ultra-processed foods have been identified as risk factors. A pooled analysis of three US cohorts found that participants in the highest quintile of ultra-processed food consumption had approximately 18–20% higher odds of developing adenomas or serrated adenomas than those with minimal intake (OR 1.18 for adenoma; OR 1.20 for serrated adenomas). This association remained significant after adjusting for body mass index and other dietary factors, indicating that the composite effect of highly processed, nutrient-poor foods contributes independently to adenoma and serrated adenoma formations (9).

**Table 1:** The impact of dietary patterns on the occurrence and recurrence of intestinal adenomas.

Dietary pattern	Group	Trend	Author and Year	Research type	Number	Effect value (95% CI), *p*-value	Comparison (highest group vs. lowest group)
Priori dietary pattern (MD)	Occurrence	Protective effect	Fliss-Isakov et al. (2018) (7)	Single-centre case–control study	783	0.93 (0.83–1.05), *p* > 0.05	Adenoma
						0.80 (0.72–0.89), *p* < 0.05	Advanced adenoma
	Occurrence	Protective effect	Dixon et al. (2007) (8)	Multicentre cohort study	33,971	0.79 (0.68–0.92), *p*<0.05	Adenoma (≥6 vs. ≤ 2)
						0.71 (0.56–0.89), *p*<0.05	Advanced adenoma (≥6 vs. ≤ 2)
	Recurrence	Protective effect	Cottet et al. (2005) (21)	Multicentre cohort study	442	0.30 (0.09–0.98), *p* < 0.05	Adenoma Q3 vs. Q1 (Female)
						1.04 (0.50–2.16), *p* > 0.05	Adenoma Q3 vs. Q1 (male)
Priori dietary pattern (DASH)	Occurrence	Protective effect	Dixon et al. (2004) (39)	Multicentre cohort study	33,971	0.75 (0.62–0.91), *p* < 0.05	Adenoma (≥5 vs. ≤ 2)
						0.67 (0.50–0.91), *p* < 0.05	Advanced adenoma (≥5 vs. ≤ 2)
Priori dietary pattern (ultra-processed foods)	Occurrence	Risk factors	Hang et al. (2023) (9)	Multicentre Cohort study	142,052	1.18 (1.11–1.26), *p* < 0.05	Adenoma (Q5 vs. Q1)
						1.20 (1.13–1.28), *p*<0.05	Serrated lesions (Q5 vs. Q1)
						1.17 (1.07–1.28), *p*<0.05	Advanced adenoma (Q5 vs. Q1)
Posteriori dietary pattern (processed meat and discretionary food)	Occurrence	Risk factors	van der Pols et al. (2025) (11)	Case–control study	724	2.60 (1.32–5.09), *p* < 0.05	Adenoma
						2.13 (1.13–4.00), *p*<0.05	Serrated lesions
Posteriori dietary pattern (healthy lifestyle)	Occurrence	Protective effect	Li et al. (2022) (12)	Cross-sectional study	24,480	0.82 (0.79–0.84), *p*<0.05	Adenoma
						0.73 (0.69–0.78), *p*<0.05	Serrated lesions
Posteriori dietary pattern (low-fat, high-fiber)	Recurrence	Not statistically significant	Schatzkin et al. (2000) (24)	Prospective multicentre RCT	2079	1.00 (0.90–1.12), *p*>0.05	Intervention vs. control (four-year follow-up)
Mechanism-based dietary indices (E-DII)	Occurrence	Risk factors	Sung et al. (2023) (14)	Case–control study	5,608	1.38 (1.12–1.72), *p* < 0.05	Adenoma (Q4 vs. Q1)
						1.37 (1.06–1.77), *p* < 0.05	Serrated lesions (Q4 vs. Q1)
	Occurrence	Risk factors	He et al. (2023) (15)	Case–control study	246	3.07 (1.23–8.14), *p* < 0.05	Adenoma (Q3 vs. Q1)
Mechanism-based dietary indices (DII)	Recurrence	Risk factors	Sardo Molmenti et al. (2016) (16)	Cohort study	1,727	0.9 (0.74–1.22), *p* > 0.05	Adenoma (Q3 vs. Q1)
Mechanism-based Dietary Indices (EDIP)	Occurrence	Not statistically significant	Nepal et al. (2023) (17)	Cohort study	21,192	0.95 (0.78–1.15), *p*>0.05	Adenoma (Q5 vs. Q1)
	Recurrence	Not statistically significant	Nepal et al. (2023) (17)	Cohort study	21,192	0.78 (0.57–1.08), *p* > 0.05	Adenoma (Q5 vs. Q1)
Mechanism-based Dietary Indices (Sulfur microbial)	Occurrence	Risk factors	Nguyen et al. (2021) (23)	Cohort study	59,013	1.31 (1.10–1.56), *p* < 0.05	Adenoma (Q4 vs. Q1)
Mechanism-based Dietary Indices (late eating)	Occurrence	Risk factors	Adnan et al. (2024) (20)	Cross-sectional study	663	1.98 (1.19–3.28), *p* < 0.05	Adenoma

MD (Mediterranean diet); DASH (Dietary Approaches to Stop Hypertension); E-DII (Energy-Dietary Inflammation Index); EDIP (Empirical dietary inflammatory pattern).

#### 3.1.2 A posteriori dietary patterns and the occurrence of intestinal polyps

Data-driven dietary patterns derived from factor or cluster analyses consistently associate Western diets with a higher adenoma risk and prudent diets with a protective effect. A meta-analysis of observational studies confirmed that unhealthy patterns, characterised by a high consumption of red and processed meat, refined grains, desserts, and high-fat foods, significantly increased the odds of colorectal adenomas, whereas diets rich in fruits, vegetables, and whole grains lowered the risk (10). One case–control study identified three dietary patterns and revealed a pronounced carcinoma effect of the Westernised pattern; individuals with the highest adherence to a “processed meats and convenience foods” pattern had more than double the odds of developing adenomas. This pattern was associated with an odds ratio of 2.13 (95% CI 1.13–4.00) for serrated adenoma and 2.60 (95% CI 1.32–5.09) for adenomas, compared to a diet history with no adenoma (11). In contrast, a healthy lifestyle score includes healthy dietary patterns. This comprises a higher proportion of fresh fruits and vegetables and a lower intake of red meat. It is particularly against serrated adenomas. Adherence to this pattern has been reported to be associated with a 40% lower risk of serrated adenomas than in patients with adenomas (OR 0.60, 95% CI 0.36–0.98), suggesting that a diet rich in fibre and unsaturated fats may specifically mitigate the development of serrated adenomas (12). The observations are consistent with findings from diverse populations, where meat- and fat-centric diets correlated with higher adenoma prevalence in American, European, and Asian cohorts, whereas plant-based diets were protective across all ethnic groups. The underlying mechanisms likely involve the pro-carcinogenic factors in Western diets, such as haem iron, nitrates, and saturated fat, which foster a tumour-promoting colonic environment, whereas the fermentation of dietary fibre and the anti-inflammatory properties of plant-based diets inhibit neoplastic initiation (13).

#### 3.1.3 Mechanism-based dietary indices and the occurrence of intestinal polyps

Researchers have developed indices that target specific mechanistic pathways linking diet to colorectal adenomas. An example is the Dietary Inflammatory Index (DII), which scores an individual’s diet based on its pro- or anti-inflammatory potential, as derived from the nutrient and food intake. A pro-inflammatory diet, reflected by a high DII score, is hypothesised to elevate systemic levels of cytokines and eicosanoids, such as IL-6 and TNF-*α*, thereby possibly promoting adenoma formation in the colonic epithelium. Epidemiological findings support this hypothesis; in a large colonoscopy-based study (*n* > 5,000), participants with adenomas, consuming the most pro-inflammatory diets, had a 38% increase in the odds of colorectal adenomas than those with the most anti-inflammatory diets (OR 1.38, 95% CI 1.12–1.72), and participants with serrated adenomas had a 37% increase (OR 1.37, 95% CI 1.06–1.77) (14). Similarly, a high DII score was associated with a higher risk of serrated adenoma. Smaller studies also concur; for instance, a case–control study in China found that participants with a more pro-inflammatory diet had significantly higher odds of colorectal adenomas, even after adjusting for BMI, smoking, and other factors (15). Interestingly, although many studies have confirmed the association of DII with intestinal and serrated adenomas, findings from a US cohort study indicated that DII is not associated with the recurrence of intestinal adenomas (16). Furthermore, the Empirical Dietary Inflammatory Pattern (EDIP), a metric derived from foods known to influence circulating inflammatory markers, was not significantly associated with the risk of adenoma in the PLCO cohort, although it was so with the risk of adenoma recurrence (17). Given these inconsistent results, more rigorous cohort studies would be required to clarify the relationship between inflammatory dietary patterns and adenomas.

Furthermore, diets can be indexed based on their effects on the metabolic and microbial pathways implicated in colorectal adenomas. The sulphur microbial diet is a recently described pattern that reflects a high intake of sulphur-rich proinflammatory foods that foster sulphur-metabolising bacteria in the gut. This index, which scores the diets high in processed meats and low in vegetables, has been shown to predict early-onset adenomas. In the Nurses Health Study II, women under 50 in the highest quartile of sulphur microbial diet score had 31% greater odds of developing an adenoma relative to those in the lowest quartile (OR: Q4/Q1 = 1.31). Importantly, this association was specific to conventional adenomas, with no increase in the risk of serrated adenoma observed with a sulphur-rich diet pattern. The dietary effect was pronounced for the most aggressive precursors, nearly doubling the odds of advanced adenomas with villous features (OR 1.65; tubulovillous/villous histology). These findings suggested a mechanistic link among a meat-centric, low-fibre diet, expansion of H2S-producing gut microbes, and early neoplastic changes in the colonic mucosa (18, 19).

Hyperinsulinemic dietary patterns, typically high in rapidly absorbable carbohydrates, sugary drinks, and red meat, but low in fibre, can drive chronic insulin secretion and insulin resistance. This metabolic state may promote colorectal polyps by upregulating IGF-1 and related growth pathways in the colonic cells. Although formal “Dietary Insulin Index” scores are still being refined, diets with high glycaemic load and poor insulin response profile have been linked to colorectal polyps. For example, large cohort studies have associated an empirical hyperinsulinemic diet pattern, rich in red and processed meats, refined grains, and sweet desserts, with an elevated risk of CRC. Such diets are therefore likely to increase the risk of adenoma. Indeed, many foods that comprise this “insulinogenic” pattern overlap with the Western diets known to raise polyp incidence. Prospective data on insulin-indexed diets and adenomas are emerging now; one study found that an insulinemic pattern score did not predict adenoma risk in a screening population, although the null finding could reflect limitations in index derivation or cohort differences. Further studies would be required to examine the composite dietary scores for insulin load and their relationship with adenoma recurrence in patients with metabolic syndrome (17).

Finally, the timing and rhythmic pattern of food intake has emerged as a novel factor in the development of colorectal adenoma. Irregular eating patterns that disrupt circadian homeostasis may promote tumorigenesis through hormonal and metabolic imbalances. A recent cross-sectional study identified late-night eating as a potential risk factor for colonic adenomas. The association persisted after controlling for diet quality and lifestyle, with late eaters exhibiting nearly two-fold increased odds of tubular adenomas (OR = 1.98). Moreover, late eating was independently associated with advanced adenomas. Although evidence specific to serrated lesions is currently lacking, chronically mistimed eating could plausibly influence all polyp pathways via systemic inflammation and metabolic stress (20).

#### 3.1.4 Strength of evidence for dietary patterns in polyp recurrence

Overall, there is relatively limited evidence regarding dietary patterns and the recurrence of intestinal polyps, and conclusions are inconsistent. *A priori* dietary patterns and mechanism-based dietary patterns have been shown to be associated with the recurrence of intestinal polyps, but the level of evidence is low. In a European intervention trial, patients who adhered most closely to the Mediterranean diet developed significantly fewer new polyps over 3 years. Among women in this trial, those in the highest tertile of the Mediterranean pattern score had 70% lower odds of polyps recurrence than those in the lowest tertile (OR 0.30, 95% CI 0.09–0.98, *p* < 0.05) (21). Interestingly, the relationship between dietary inflammation and the recurrence of intestinal polyps remains controversial. In the PLCO cohort, EDIP was shown to be negatively correlated with polyps recurrence (17). The study suggested that DII is not associated with polyp recurrence (17). The evidence level is relatively low owing to the small number of participants in cohorts and the limited generalizability of the findings. Studies with relatively high levels of evidence suggest that a posteriori dietary patterns as an intervention are not associated with the recurrence of intestinal polyps. For instance, the European Fiber and Calcium Intervention trial found no significant link between a high-fat, high-sugar pattern and polyp recurrence over 3 years (22). A larger prospective, multicentre, randomised controlled trial assigned participants either to receive a dietary education promoting a low-fat, high-fibre pattern or to receive no such education. Follow-up at 4 and 8 years demonstrated that dietary education was ineffective in reducing the recurrence rate of colorectal adenomas. Interestingly, without changing the main conclusions, a further analysis of the intervention group at the 4-year follow-up revealed that adherence to a low-fat, high-fibre diet was associated with a significantly lower rate of intestinal polyp recurrence (23–25). However, it is important to note that the compliance analysis should only be considered exploratory, and the primary conclusion remains negative.

### 3.2 Dietary patterns and CRC

#### 3.2.1 Priori dietary patterns

While some studies have indicated that the association between Mediterranean dietary pattern and CRC incidence is not statistically significant (26, 27), meta-analyses have indicated that adherence to a high-Mediterranean dietary pattern corresponds to an approximate 15–17% reduction in CRC risk. The latest review by the World Cancer Research Fund (WCRF) expert panel rated the evidence as limited, suggesting that greater alignment with healthy *a priori* patterns (Mediterranean type, healthy plant-based indices, and DASH) is associated with reduced CRC incidence (28). These protective diets commonly feature a high intake of fibre-rich plant foods and moderate dairy consumption, along with limited red and processed meats. Epidemiological evidence shows that *a priori* dietary patterns exhibit broad consistency across different populations. Cohort studies in North America, Europe, and Asia have generally reported an inverse association between higher diet quality scores and CRC risk, with little heterogeneity by geographic region or sex in most analyses. For instance, in pooled analyses, men and women in the highest quartile of DASH scores experienced reductions in CRC risk similar to those with poorer dietary scores. Nevertheless, some sex-specific differences were observed in certain dietary patterns. Adherence to a Mediterranean diet is associated with a significant reduction in CRC risk among men, whereas the evidence for women is weaker in some cohorts. A large European study found that a high Mediterranean pattern score was linked to a 20% lower CRC risk in men but no significant protective effect in women. These disparities might reflect differences in baseline diet, hormonal factors, or interactions with the gut microbiome between the sexes. Based on anatomical subsite, healthy diets typically confer stronger protection to the distal colon and rectum. For example, Mediterranean-style eating shows a modest association with reduced rectal cancer risk (often 20–30% lower) but has little effect on the proximal colon (26, 27, 29–36) (Table 2).

**Table 2:** The impact of dietary patterns on the colorectal cancer.

Dietary pattern	Trend	Author and Year	Research type	Number	Effect value (95% CI), *p*-value	Comparison (highest group vs. lowest group)
Priori dietary pattern (MD)	Not statistically significant	Schulpen et al. (2020) (26)	Cohort study	120,852	1.02 (0.90–1.17), *p*>0.05	6.00–9.00 vs. 0.00–3.00
	Not statistically significant	Lavalette et al. (2018) (27)	Cohort study	41,543	1.02 (0.51, 2.04), *p*>0.05	Q5 vs. Q1
	Not statistically significant	Petimar et al. (2018) (29)	Cohort study	78,012	0.99 (0.83–1.18), *p*>0.05	≤9 vs. ≥ 0
	Protective effect	Park et al. (2017) (30)	Multicentre Cohort study	190,449	0.89 (0.80–0.99), *p*<0.05	6–9 vs. 0–2
	Protective effect	Bamia et al. (2013) (31)	Cohort study	5,296,617	0.89 (0.80–0.99)	7–9 vs. 0–3
	Not statistically significant	Jones et al. (2017) (32)	Cohort study	35,372	0.82 (0.55–1.24), *p*>0.05	Q5 vs. Q1
	Protective effect	Reedy et al. (2008) (33)	Cohort study	492,382	0.87 (0.68–0.98)	Q5 vs. Q1
Priori dietary pattern (DASH)	Protective effect	Nguyen et al. (2020) (34)	Cohort study	132,606	0.90 (0.78–1.03), *p*<0.05	Q4 vs. Q1
	Not statistically significant	Petimar et al. (2018) (29)	Cohort study	78,012	0.89 (0.74–1.08), *p*>0.05	Q5 vs. Q1
	Protective effect	Park et al. (2017) (30)	Multicentre Cohort study	190,949	0.80 (0.72–0.88), *p* < 0.05	Q5 vs. Q1
	Protective effect	Miller et al. (2013) (35)	Cohort study	491,841	0.78 (0.72–0.84)	Q5 vs. Q1
	Protective effect	Vargas et al. (2016) (36)	Cohort study	911	2.60 (1.32–5.09), *p* < 0.05	30–60 vs. 8–20
Priori Dietary Pattern (ultra-processed foods)	Risk factors	Meine et al. (2024) (37)	Cohort study	415,434	1.11 (1.03–1.21), *p*<0.05	Q4 vs. Q1
Posteriori Dietary Pattern (vegetable pattern)	Not statistically significant	Dixon et al. (2004) (39)	Multicentre Cohort study	211,448	1.03 (0.83–1.19)	Q4 vs. Q1
Posteriori Dietary Pattern (vegetable–fruit–soy pattern)	Not statistically significant	Butler et al. (2008) (40)	Cohort study	61,321	1.02 (0.83–1.24)	Q4 vs. Q1
Posteriori Dietary Pattern (low-fat, high-fibre)	Not statistically significant	Mehta et al. (2017) (41)	Cohort study	13,217	0.90 (0.78–1.02)	Q4 vs. Q1
Posteriori Dietary Pattern (prudent diet)	Not statistically significant	Shin et al. (2018) (42)	Cohort study	43,469	0.90 (0.78–1.02)	Q5 vs. Q1
Posteriori dietary pattern (prudent diet)	Protective effect	Mehta et al. (2017) (41)	Cohort study	137,217	0.78 (0.64–0.96), *p* < 0.05	Q4 vs. Q1
Posteriori Dietary Pattern (high-dairy, high-fruit-and-vegetable, low-alcohol pattern)	Protective effect	Kumagai et al. (2014) (43)	Cohort study	44,097	0.76 (0.60–0.97), *p* < 0.05	Q4 vs. Q1
Mechanism-based Dietary Indices (EDIH)	Risk factors	Yue et al. (2021) (44)	Cohort study	94,217	1.67 (1.15–2.44), *p* < 0.05	0.50–4.80 vs. −1.40 to −0.30
	Risk factors	Tabung et al. (2018) (45, 46)	Cohort study	46,210	1.33 (1.11–1.61), *p*<0.05	Q5 vs. Q1
	Risk factors	Tabung et al. (2018) (45, 46)	Cohort study	74,191	1.22 (1.03–1.45), *p*<0.05	Q5 vs. Q1
Mechanism-based Dietary Indices (EDIP)	Risk factors	Tabung et al. (2018) (45, 46)	Cohort study	46,804	1.44 (1.19–1.74), *p*<0.05	1.14 vs. −1.20
	Risk factors	Tabung et al. (2018) (45, 46)	Cohort study	74,246	1.22 (1.02–1.45)	1.16 vs. −1.20
Mechanism-based Dietary Indices (DIS)	Risk factors	Byrd et al. (2020) (47)	Cohort study	453,465	1.21 (1.14–1.29)	Q5 vs. Q1
Mechanism-based Dietary Indices (sulfur microbial diet score)	Risk factors	Wang et al. (2021) (48)	Cohort study	3,217	1.27 (1.12–1.44), *p*<0.05	Q5 vs. Q1

MD (Mediterranean diet); DASH (Dietary Approaches to Stop Hypertension); E-DII (Energy-Dietary Inflammation Index); EDIP (Empirical dietary inflammatory pattern); DIS (Dietary inflammation scores); and EDIH (Empirical Dietary Index for Hyperinsulinemia).

Compared to a previously defined healthy dietary pattern, a recent meta-analysis of ultra-processed foods and CRC, incorporating four large cohort studies, indicated that higher-intake groups exhibited an 11% increased risk of CRC. Further subgroup analyses indicated that a higher intake of ultra-processed foods is associated with rectal cancer incidence rather than the incidence of colon cancer. This finding suggests the need for further evidence to validate the relationship with colon cancer (37).

#### 3.2.2 Posteriori dietary patterns

A posteriori dietary patterns have been consistently identified across numerous cohorts. Large prospective studies have consistently reported that adherence to a prudent diet is associated with a lower incidence of CRC, whereas adherence to a Western diet is associated with a higher risk. One umbrella review of prospective studies found that greater adherence to “healthy” dietary patterns corresponded to an approximately 8% reduction in the risk of gastrointestinal cancer, whereas Western-style patterns conferred an approximately 14% increased risk. Similarly, a recent systematic review observed that virtually all studies identified a protective variant of a healthy pattern against CRC, particularly colon cancer, and a meat- and fat-heavy Western pattern associated with an elevated risk. These associations are generally moderate in magnitude; for instance, individuals with strong adherence to a Western diet may have a 20–30% higher CRC risk than those with minimal Western dietary features, whereas close adherence to a prudent pattern might confer a 15–20% lower risk than adherence to the least healthy diets, although estimates vary across studies and subpopulations (38).

Despite the general consistency, strength of the association for a posteriori pattern is less pronounced and more variable than that for *a priori* indices. A 2025 global systematic review concluded that the evidence linking data-driven dietary patterns to CRC risk remains limited and inconclusive. This review observed no clear dose–response relationship or consistent effect across all studies for Western or meat-centric patterns, with the pooled evidence being heterogeneous. The lack of a uniform association for the “Western” pattern was somewhat surprising, given the known risks of its components, such as processed meat, and contrasted with the earlier meta-analyses that reported significant positive associations (28). Methodological variation offers a potential explanation, since different studies derive slightly different patterns and use divergent labels, complicating direct comparisons. Furthermore, in some populations, a single “Western” factor may not fully capture all unhealthy eating behaviors, or multiple unhealthy patterns might coexist. For example, distinct “meat-rich” and “drinking” patterns can emerge, each contributing to the risk of CRC in different ways. The global systematic review grouped Western, high-meat, and high-alcohol consumption patterns together and found overall null or inconsistent results, suggesting that no single factor consistently dominated the association. Conversely, a trend was observed whereby prudent or vegetable-rich patterns were associated with a lower CRC risk in certain subgroups, particularly among men, and in specific cultural contexts, although the trend was not sufficiently uniform to constitute probable evidence. For instance, a vegetable-rich prudent diet showed clear protective effects in some Western populations and male cohorts while demonstrating weak effects in others (39–43).

#### 3.2.3 Mechanism-based dietary indices

Accumulating evidence indicates that hyperinsulinemic and pro-inflammatory dietary patterns are robustly associated with a higher risk of CRC. A comprehensive meta-analysis of 28 studies involving over 2.2 million participants found that individuals with the most pro-inflammatory diets had an approximately 39% greater risk of developing CRC than those with the most anti-inflammatory diets (pooled effect size 1.39, 95% CI 1.29–1.51). This analysis further revealed significantly elevated risks for colon cancer subsites, with relative risks of approximately 1.40 for overall colon cancer, 1.28 for the proximal colon, and 1.50 for the distal colon, when comparing the highest to the lowest dietary inflammatory scores. Similarly, high adherence to the Empirical Dietary Index for Hyperinsulinemia (EDIH), characterised by a high intake of red meat and processed foods, has been linked to a 20–44% increase in CRC incidence across multiple cohort studies. High EDIH scores yielded comparable risk elevations, reflecting the substantial overlap between an insulinogenic diet and an inflammatory diet, since EDIH and Empirical dietary inflammatory pattern (EDIP) scores were correlated at approximately 0.5–0.6. These associations persisted after adjusting for body mass index and other confounders, suggesting that diet-driven metabolic and inflammatory changes, rather than adiposity alone, contribute to colorectal carcinogenesis. The DII, applied to diverse populations worldwide, also shows a consistent relationship whereby individuals on pro-inflammatory diets experience a significantly elevated CRC risk. Prospective studies from the United States, Europe, and Asia have reported that higher DII scores are associated with a 20–60% increase in CRC risk. A 2022 meta-analysis focusing on DII found that individuals in the highest DII category had approximately 1.3-fold higher odds of developing CRC than those in the lowest category. Collectively, the evidence strongly implicates dietary inflammation and insulin response as important drivers of CRC risk (44–48).

Such pattern–disease links appear relatively consistently across population subgroups. Both men and women with high EDIP and EDIH scores exhibit higher incidences of colon cancer. However, an intriguing sex-specific finding for rectal cancer, as analysed from the Nurses’ Health Study and the Health Professionals Follow-up Study, suggested that a proinflammatory or hyperinsulinemic diet remarkably increases rectal cancer risk in men but not in women (28). In men, high EDIP and EDIH scores were associated with a 1.7-fold higher risk of rectal cancer, whereas in women these dietary patterns showed no significant association. This divergence could be explained by hormonal or metabolic differences; for instance, oestrogen in premenopausal women exerts anti-insulin and anti-inflammatory effects that might mitigate diet-induced metabolic stress, or women’s gut microbiomes might respond differently to dietary composition. Moreover, the lower absolute risk of rectal cancer in women could possibly make the detection of dietary effects more challenging. In addition to sex, the association between proinflammatory diet and CRC has been observed across various ethnic groups and geographic regions as well, implying its biological generalisability.

## 4 Discussion

Overall, while the evidence for polyp recurrence remains comparatively limited and heterogeneous, available literature suggests that dietary patterns are associated with the polyp-to-carcinoma sequence. In general, adherence to healthier *a priori* dietary patterns (such as MD and DASH) and lower inflammatory dietary profiles tends to be associated with a lower risk of incident polyps and CRC, whereas adherence to dietary patterns characterised by higher intakes of red/processed meats and refined carbohydrates is more often associated with higher risks.

Our synthesis is broadly concordant with, and extends, the findings of recent meta-analyses and large cohort studies by integrating evidence across polyp occurrence, recurrence, and CRC within a unified framework (49, 50). With respect to polyp recurrence, the current evidence is best interpreted as suggestive rather than conclusive. A few cohort analyses have reported associations between MD or EDIP and adenoma recurrence (21, 22), but these findings are limited by the small sample size. The relationship between dietary pattern and the recurrence of intestinal polyps remains controversial. Randomised trials evaluating interventions (low-fat, high-fibre dietary education) have generally shown no clear benefit in primary intention-to-treat analyses. In the adherence-based analysis, positive results were observed; however, signals observed in adherence-based analyses should be interpreted cautiously because they are vulnerable to selection bias and may reflect healthier behaviors among highly adherent participants rather than a definitive effect of diet (23, 24). Notably, results of the compliance analysis should be considered exploratory findings only and do not affect the primary negative conclusion. Notably, significant heterogeneity exists in a posteriori dietary patterns; some results regarding post-dietary interventions and intestinal polyps or CRC showed no statistical significance, potentially contributing to false negative and inconsistent findings. Future research on polyp recurrence should include adequately powered preregistered trials and well-designed randomised controlled trials.

The robust links between dietary patterns and colorectal neoplasia are biologically plausible, with chronic inflammation representing a key mechanism. Western diets, which are potently proinflammatory, elevate the levels of circulating inflammatory markers and cytokines remarkably. Insulin resistance is also closely related to carcinoma, because diets with a high glycaemic load and excessive sugars or refined grains can drive hyperinsulinemia and increase insulin-like growth factor (IGF) activity (51). The hormonal changes encourage cellular proliferation and inhibit apoptosis in the colonic epithelium, thereby fostering an environment conducive to polyp development (52). Conversely, prudent diets, such as the Mediterranean pattern, may improve insulin sensitivity and reduce subclinical inflammation, which is reflected in the lower levels of protumourigenic growth factors and oxidative stress markers among the adherents (53). The gut microbiome likely mediates many diet–cancer relationships, where low red meat intake reduces exposure to nitroso compounds and secondary bile acids that can damage colonocytes (54). In contrast, Western diets can perturb the microbiome in favour of bacteria that produce harmful metabolites, contributing to DNA damage and epithelial proliferation (55). In summary, dietary patterns appear to modulate colorectal neoplasia through multiple converging mechanisms that together influence the trajectory from normal mucosa to polyps and cancer (56).

The current review has several strengths, including a holistic perspective that integrates evidence from benign adenomas with cancer outcomes. As this study considers both polyp formation and recurrence along with cancer incidence, it offers a more complete narrative of how dietary patterns affect the polyp-to-carcinoma sequence. Additionally, the current review compared whether conclusions were consistent across different genders, ethnicities, and pathological types, enhancing the generalizability of the results. Despite these strengths, the limitations of this review should also be acknowledged. First, most evidence was derived from observational studies, which are prone to residual confounding and cannot prove causation. Individuals adhering to prudent diets may engage in other healthy behaviors that are difficult to fully consider analytically. Second, dietary-pattern scores exhibited heterogeneity, because different studies employed non-uniform dietary assessment tools, possibly introducing potential biases in result interpretation. Third, dietary-pattern evaluation relied on the use of retrospective questionnaires, which is inevitably subject to recall bias. Fourth, most studies included in this review involved community-based populations and employed colonoscopy procedures of variable quality. Future prospective cohort studies should incorporate consistent dietary-pattern scoring, adequate control for confounding factors, and well-designed subgroup analyses. Fifth, research on younger populations remains limited and warrants further investigation.

In conclusion, this review suggests that dietary patterns are associated with the polyp-to-carcinoma sequence. The adherence to recommended *a priori* dietary patterns, a posteriori dietary patterns, and mechanism-based dietary indices may have positive implications for the prevention of CRC across the entire disease course. The findings of this review provide a theoretical foundation for further efforts towards the comprehensive prevention and control of CRC.
